# Improvement of Sensing Performance of Impedancemetric C_2_H_2_ Sensor Using SmFeO_3_ Thin-Films Prepared by a Polymer Precursor Method

**DOI:** 10.3390/s19040773

**Published:** 2019-02-13

**Authors:** Tomohisa Tasaki, Satoko Takase, Youichi Shimizu

**Affiliations:** Department of Applied Chemistry, Kyushu Institute of Technology, 1-1 Sensui-cho, Tobata, Kitakyushu 804-8550, Fukuoka, Japan; takky0726@gmail.com (T.T.); satoko@che.kyutech.ac.jp (S.T.)

**Keywords:** perovskite-type oxide, thin-film, polymer precursor, acetyl acetone, gas sensor, AC impedance, acetylene

## Abstract

A sensitive an impedancemetric acetylene (C_2_H_2_) gas sensor device could be fabricated by using perovskite-type SmFeO_3_ thin-film as a sensor material. The uniform SmFeO_3_ thin-films were prepared by spin-coating and focusing on the effects of polymer precursor solutions. The prepared precursors and thin-films were characterized by means of thermal analysis, Fourier-transform infrared spectroscopy, ultraviolet–visible spectroscopy, X-ray diffraction analysis, scanning electron microscopy and X-ray photoelectron spectroscopy. It was found that particle growth and increase in homogeneity of the prepared thin-film could be accelerated by the addition of acetyl acetone (AcAc) as a coordination agent in the polymer precursor solution. Moreover, the highly crystallized thin-film-based sensor showed good response properties and stabilities to a low C_2_H_2_ concentration between 0.5 and 2.0 ppm.

## 1. Introduction

Hydrocarbon gases are mostly toxic and flammable; for example, automobile exhaust and industrial plant soot contain carbon monoxide (CO), nitrogen oxide (NO_X_) and hydrocarbons (HCs) which cause various environmental issues. In the agro-food field, ethylene (C_2_H_4_) is known as a growth hormone for vegetables and fruits [1,2]. C_2_H_2_, the detection gas in this study, is also an important industrial gas, which has been used in the field of synthetic chemistry as a starting material of benzene and poly-acetylene. Recently, it is known that C_2_H_2_ is generated from the insulating oil of transformers when the oil becomes deteriorated. Therefore, a C_2_H_2_ sensor should be used for on-site monitoring of the transformer, in spite of the generated C_2_H_2_ having a low concentration of 0.5 ppm or lower [3,4,5]. Thus, studies about C_2_H_2_ sensors have recently increased in number due to the importance of C_2_H_2_ detection. 

Perovskite-type oxides, which are functional inorganic materials, have a wide range of applications and various interesting properties, including ion conductivity [6] and catalytic activity [7]. The perovskite-type oxide is also used as a good sensor material, and has displayed sensing properties toward various harmful gases, such as NO_X_ [8], VOC [9], NH_3_ [10], CO [11] and hydrocarbons [12]. 

In this study, we focused on a semiconductive gas sensor based on a perovskite-type oxide, because of its good sensitivity and long stability. In the sensing function of a semiconductive gas sensor using oxide materials, it is important to discuss chemical adsorption over the oxide surface, because sensor response shows a similar behavior to a catalyst. Many research results of density functional theory of CO adsorption properties for Fe-based perovskite oxides have been reported, and this way of thinking could improve the sensor properties because the electron orbital state (HOMO–LUMO) of B-site metal of the BO_6_ octahedral ion influences many chemical properties [13,14,15]. In fact, we have reported that the SmFeO_3_ thin-film is the only sensor material to have high sensitivity and selectivity to C_2_H_2_ among the prepared SmBO_3_ (B=Cr, Mn, Fe, Co) thin-films [16,17]. Therefore, we are currently researching the response mechanism from a standpoint of HOMO–LUMO energy interaction between the oxide and C_2_H_2_.

By the way, the use of an oxide thin-film would give higher sensitivity and performance, and there are many ways to obtain a homogeneous and good crystalline thin-film [18,19,20,21,22,23]. 

In this study, we tried to synthesise perovskite-type thin-films by a wet-chemical route using a polymer precursor method prepared by adjustment of the additive amount of AcAc as a coordination agent in the precursor. The C_2_H_2_ sensing properties of the devices and the mechanism were discussed. We have developed a highly sensitive C_2_H_2_ sensor device.

## 2. Materials and Methods

### 2.1. Preparation and Analysis of Precursor and Oxide Thin-Film

**Figure 1:**
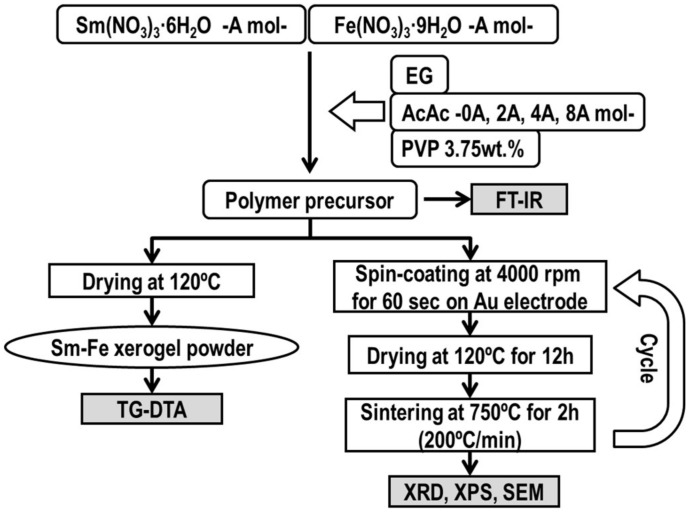
Flowchart for preparation route of perovskite-type oxide SmFeO_3_ thin-films.

Figure 1 shows the process of preparation of the perovskite-type oxide SmFeO_3_ thin-film by a polymer precursor method, in which stoichiometric amounts of metal nitrates, Sm(NO_3_)_3_·6H_2_O and Fe(NO_3_)_3_·9H_2_O (0.5 mol + 0.5 mol = A mol) were dissolved in ethylene glycol (EG; 5 mL) solvent. Then, multiples of 2-, 4- and 8-times the solvent amount of acetyl acetone (AcAc; 2A, 4A and 8A mol, respectively) and polyvinylpyrrolidone (PVP; 3.75 wt % of total materials) as a coordination agent and a polymer additive, respectively, were added to this solution. The solutions were abbreviated as AcAcX, in which X is the mol content of acetyl acetone (X = 0, 2, 4, 8). The AcAc0 means a solution without AcAc. The prepared precursor solutions were spin-coated on an alumina substrate with Au interdigitated electrodes at 4000 rpm, and finally sintered at 750 °C for 2 h in air. The spin-coating and sintering processes were repeated 3 times [16] to avoid making cracks.

Characterizations of the prepared materials were carried out by means of the following methods; Fourier-transform infrared spectroscopy (FT-IR, IRPrestige-21, Shimadzu, Kyoto, Japan), ultraviolet–visible spectroscopy (UV-Vis, U-3900, Hitachi, Kyoto, Japan), thermogravimetric–differential thermal analysis (TG-DTA, TG-8120, Rigaku, Japan), and X-ray diffraction analysis (XRD, JDX3500K, JEOL, Kyoto, Japan) using CuKα radiation. Surfaces of the thin-films were analyzed by a scanning electron microscope (SEM, JSM-6701, JEOL, Kyoto, Japan) and X-ray photoelectron spectroscopy (XPS, Axis-Nova, Shimadzu Kratos, Kyoto, Japan) using Al Kα radiation, and all binding energy values were calibrated with the C_1s_ line at 284.5 eV.

### 2.2. Measurement of Sensing Properties

**Figure 2:**
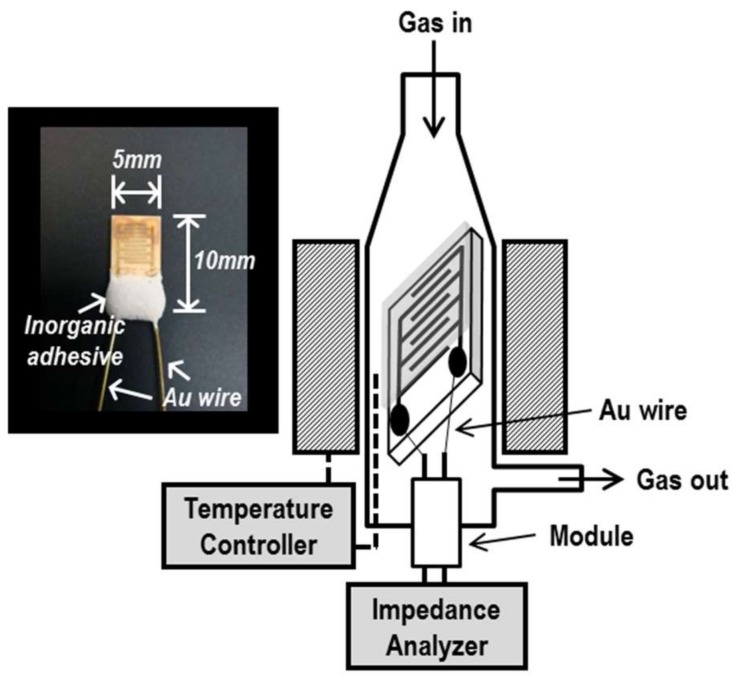
Schematic diagrams of the measurement apparatus and the actual image of the oxide thin-film device.

The measurement apparatus and the sensor device are shown in Figure 2. The sensor device was attached to a gold wire (*ϕ* = 0.3 mm) with Ag paste and connected to an LCR meter (HIOKI 3532-50). Gas sensing properties of the device were investigated by an AC impedance method with an applied voltage of 0.5 V at the frequency range between 50 Hz and 5 MHz. Measurement temperatures were used between 300 and 500 °C. Gas concentration was controlled by mixing with dry synthetic C_2_H_2_ + N_2_ parent gas mixture, dry synthetic air (N_2_ and O_2_), N_2_, and O_2_ at a fixed concentration of $P_{o_{2}}$= 0.21 atm under a gas flow rate of 100 cm^3^/min.

## 3. Results and Discussion

### 3.1. Preparation of the Precursor Solutions

**Figure 3:**
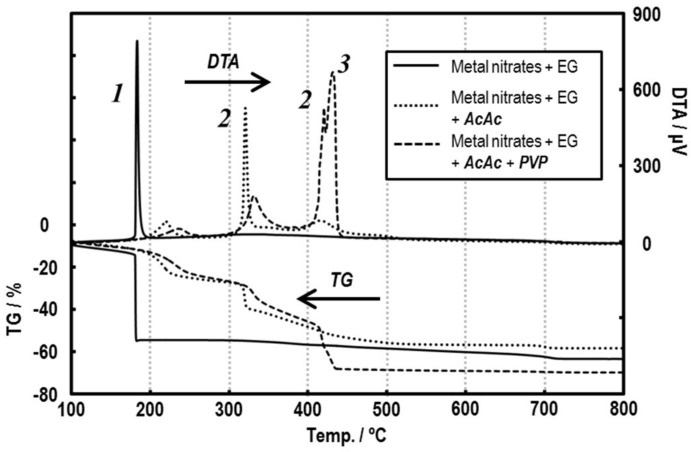
TG–DTA curves of Sm–Fe precursors dried at 120 °C.

To investigate effects of AcAc addition for the precursor, thermal behavior was firstly measured. Figure 3 shows TG–DTA curves of the precursor powders prepared by drying at 120 °C. In the case of the AcAc0 powder, the DTA curve showed a large exothermic reaction with a weight loss (ca. 40%) at ca. 180 °C, which corresponds to the pyrolysis of remaining EG and nitrates of the starting materials. However, all DTA curves of precursors with AcAc indicated different exothermic reactions, that is, a weight loss (ca. 10%) was indicated at ca. 210 °C instead of diminishing the pyrolysis at 180 °C. This reaction might be due to a pyrolysis of the excess amounts of AcAc. Moreover, the TG–DTA curves showed a large exothermic peak with 10% weight loss at ca. 320 °C, leaving a complex of EG and AcAc linked like a polymer with metal cations formed in the precursor under heat conditions [21]. There is, furthermore, a large pyrolysis divided into two peaks at 420–440 °C. Both reactions could be assigned to the decomposition of the complex network and the combustion of PVP, respectively.

**Figure 4:**
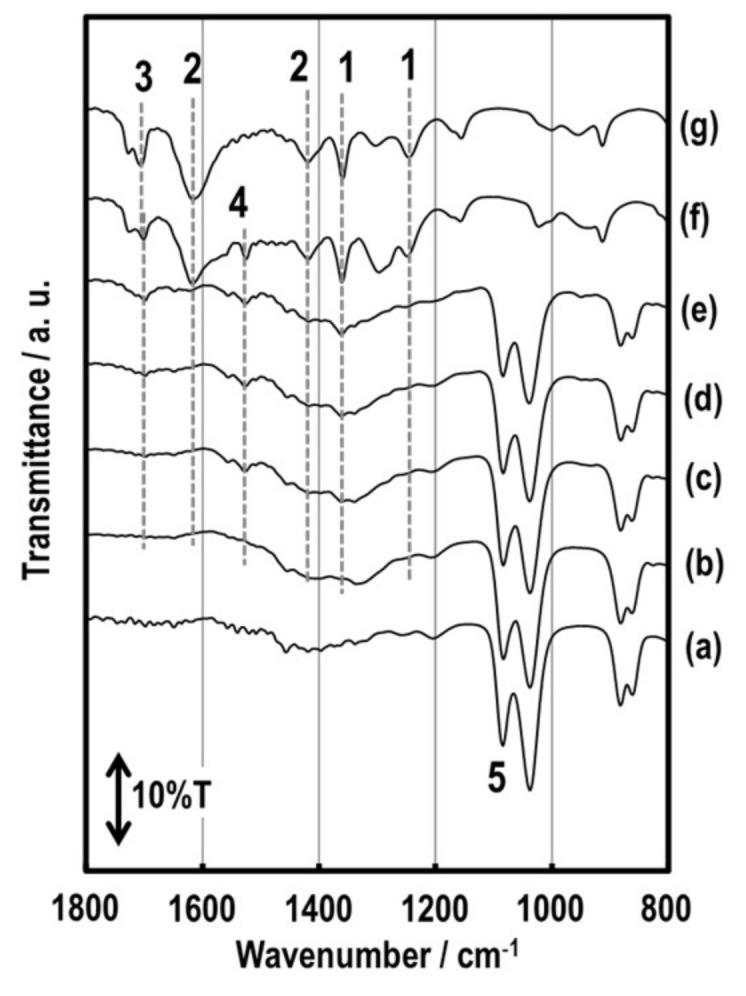
FT–IR spectra of the various solutions and the Sm–Fe precursor solutions without PVP; (**a**) EG only, (**b**) AcAc0, (**c**) AcAc2, (**d**) AcAc4, (**e**) AcAc8, (**f**) metal nitrates with AcAc, and (**g**) AcAc only.

FT–IR spectra of the solutions are shown in Figure 4. The peak #3, which could be assigned to the presence of a carboxylic (C=O) stretch peak from AcAc, appears at 1705 cm^−1^ in Figure 4g [19,23]. In Figure 4f, the peak that shifted from 1705 cm^−1^ to 1697 cm^−1^ could seem to be a link between metal cations and carboxylic oxygen, and this brought about an increase in the moment of the bond from the relation between energy and wavelength [19]. Peak #4 at 1523 cm^−1^ appeared after adding metal nitrates in AcAc, which could be attributed to the bond between both materials [24]. The peaks #1 at 1242 and 1357 cm^−1^ and the peaks #2 at 1415 and 1612 cm^−1^ were attributed to the asymmetric vibration of the carbon chain of AcAc and C=O stretching modes, respectively. The two strong peaks #5 at 1037 and 1083 cm^−1^ also corresponded to C–O stretching of primary and secondary alcohols [22]. Additionally, these phenomena could be corroborated by UV–Vis spectra due to a broad peak for a d–d electron transition and a shoulder peak for a charge-transfer transition at ca. 450 and 350 nm, respectively.

### 3.2. Structure of the SmFeO_3_ Thin-Films

**Figure 5:**
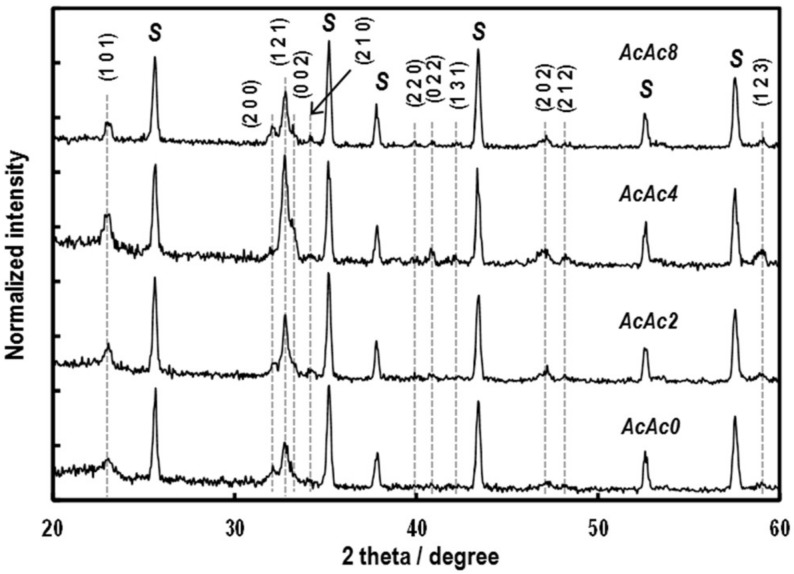
XRD patterns of the SmFeO_3_ thin-films prepared from AcAc0, 2, 4 and 8 precursors at 750 °C.

**Figure 6:**
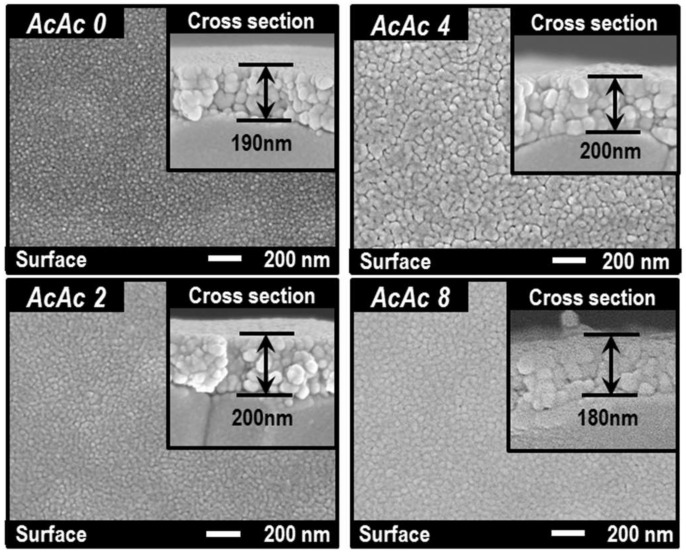
SEM images of the SmFeO_3_ thin-films prepared from AcAc0, 2, 4 and 8 precursors at 750 °C.

Figure 5 shows XRD patterns of oxide thin-films sintered at 750 °C for 2 h. The oxide thin-films showed almost single-phase perovskite-type SmFeO_3_, and the peak intensities increased with increasing the amounts of AcAc. It could be seen that AcAc accelerates crystal growth of the oxides. Figure 6 shows SEM images of the SmFeO_3_ thin-films prepared by AcAc0, 2, 4 and 8 precursors sintered at 750 °C. We can see an increase in particle size of the SmFeO_3_ thin-film with increasing amounts of AcAc. Particles of the AcAc8 film seemed to clump together, although grain size of the AcAc8 film was smaller than that of the AcAc4 film. However, there is no large difference in film thickness.

### 3.3. Sensing Properties of the SmFeO_3_ Thin-Film Devices

**Figure 7:**
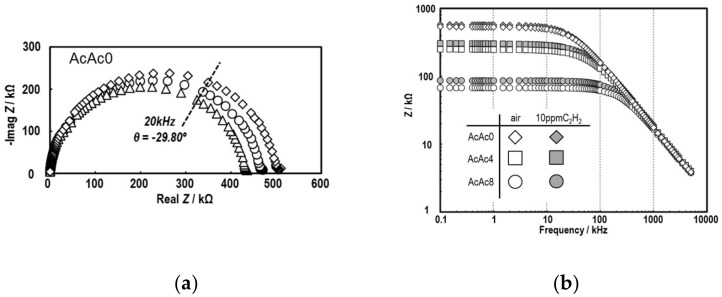
Nyquist plot in air (**a**) and Bode diagram in air and 10 ppm C_2_H_2_ (**b**) of the SmFeO_3_ device prepared from AcAc0, 4 and 8 precursors at 400 °C.

**Figure 8:**
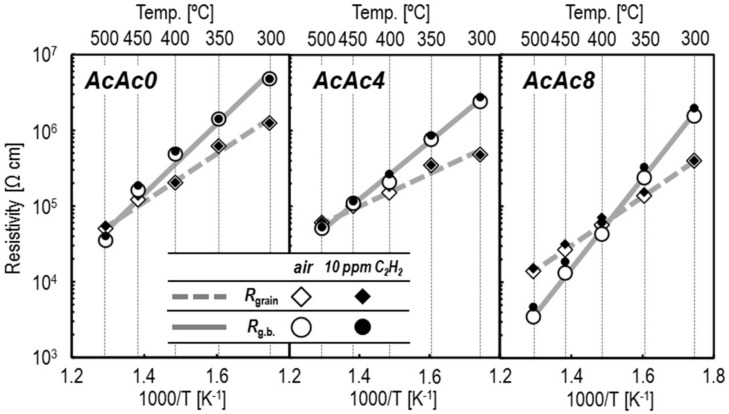
Temperature dependencies of resistivity of grain and grain boundary [g.b.] for the SmFeO_3_ thin-film devices at 300–500 °C.

As it is important to evaluate the frequency properties of a device in AC impedance measurement, the resistance components of a sensor using polycrystalline ceramics with bulk, grain boundary and electrode interfaces could be identified by setting a frequency. Figure 7 shows Nyquist and Bode plots of the SmFeO_3_ thin-films prepared by AcAc0, 2, 4 and 8 precursors at 400 °C. The Nyquist plots of the SmFeO_3_ thin-film by the AcAc0 precursor showed a capacitance semicircle with a negative phase angle. It could be found that the SmFeO_3_ thin-film has a p-type semiconductive property due to the fact that its impedance decreased entirely when the atmosphere was switched from P_O2_ = 0.21 atm to 0.75 atm [25]. On the other hand, the SmFeO_3_ device showed increasing impedance at 10 ppm C_2_H_2_. Bode plots of the SmFeO_3_ thin-films by AcAc0–8 precursors had some feature points. Firstly, the impedance of the device showed a constant value at a lower frequency and a stepwise decrease at a higher frequency. Secondly, the impedance of the devices decreased entirely in air by increasing AcAc. Finally, the devices with more AcAc had an increased rate of impedance to C_2_H_2_, especially at a low frequency. The reason why the thin-films with or without AcAc showed such behaviors should be that the thin-films that became more crystalline with AcAc had a lower potential among grains. To study the temperature dependencies of bulk and grain boundary resistance to C_2_H_2_, Nyquist plots from the SmFeO_3_ thin-film devices at 300–500 °C were divided from bulk and grain boundaries by fitting. The sensor resistances were plotted against measurement temperature, as shown in Figure 8. It was found that the grain boundary of the AcAc8 film showed a strong dependence on temperature, and changed resistance largely at 10 ppm C_2_H_2_. Therefore, the SmFeO_3_ thin-film from the AcAc precursor has superior properties for an AC sensing material.

**Figure 9:**
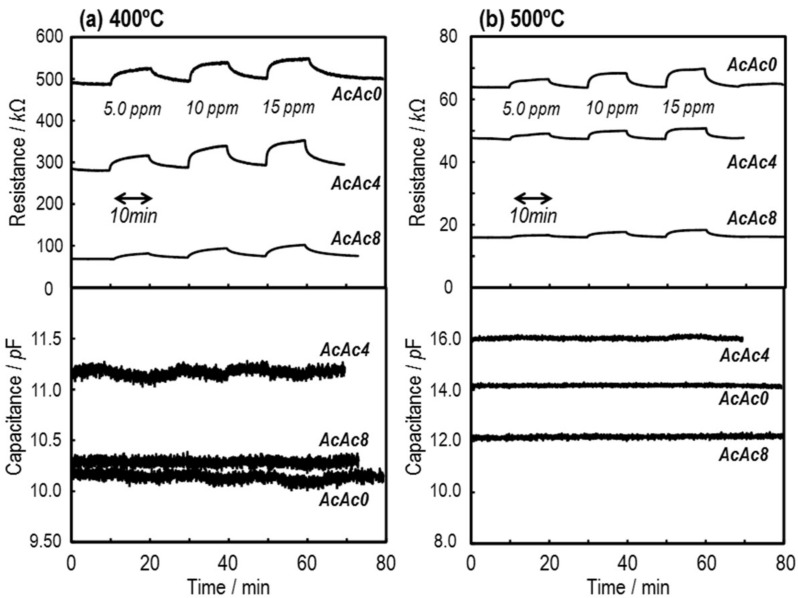
Response transients in resistance *R* and capacitance *C* components of the SmFeO_3_ thin-film devices prepared from AcAc0, 4 and 8 precursors to 5–15 ppm C_2_H_2_ at (**a**) 400 and (**b**) 500 °C at 20 kHz.

**Figure 10:**
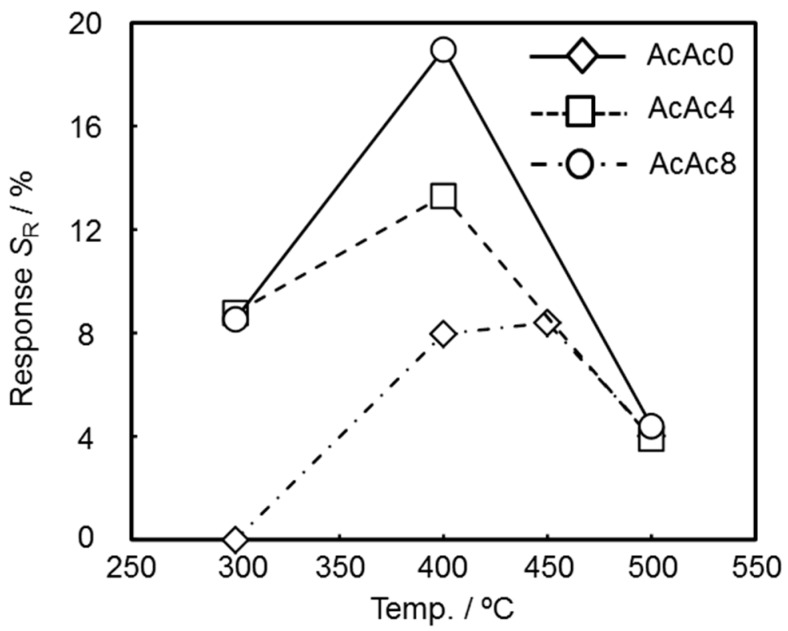
Temperature dependencies of *S*_R_ for the SmFeO_3_ devices at 5 ppm C_2_H_2_ at 20 kHz.

Figure 9 shows the responses of the SmFeO_3_ thin-film devices to 5–15 ppm C_2_H_2_ at 400 and 500 °C at 20 kHz. None of the devices detected C_2_H_2_ in capacitance *C*, but they responded clearly in resistance *R*. The reason why capacitance of the devices did not show responses might be derived from the low conductivity and effect of ambient noise. With increasing the amount of additive AcAc, *R* of the devices decreased and became more stable. Moreover, sensor response and recovery speed were improved with increasing measurement temperature. Temperature dependencies of resistance response *S*_R_ to 5 ppm C_2_H_2_ at 300–500 °C are shown in Figure 10. Here, the sensor response *S*_R_ was defined as in *S*_R_ = (*R*_gas_ − *R*_air_)/*R*_air_ × 100%, where *R* is the resistance component in air or sample gas, written in subscript as air and gas, respectively. The temperature that the maximum response was obtained at was ca. 400–450 °C, and the AcAc8 thin-film device showed the highest response. This implies that the prepared devices have roughly equal chemical activity, and gas diffusion might be working effectively due to this result [26].

**Figure 11:**
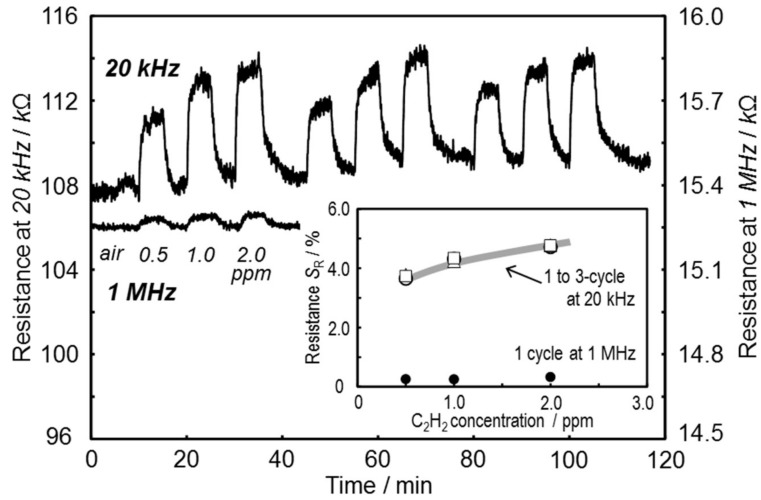
Response transient and concentration dependencies (insert) of the SmFeO_3_ thin-film device prepared from AcAc8 precursor to low C_2_H_2_ concentration at 400 °C, 20 kHz and 1 MHz.

Response transients and concentration dependencies of the SmFeO_3_ thin-film device AcAc8 at a low C_2_H_2_ concentration at 400 °C, 20 kHz are shown in Figure 11. The response properties of the device at 1 MHz, which took a bulk component to compare the results of frequency, are shown simultaneously in Figure 11. The response curve at 1 MHz showed a small resistance change of about 0.8 kΩ at 2 ppm C_2_H_2_. The response curve at 20 kHz, however, showed a larger and faster response at 0.5 ppm C_2_H_2_. According to Figure 11, the SmFeO_3_ thin-film AcAc8 indicated the ability to be a lower-C_2_H_2_-concentration sensor, because the device showed a relatively stable response with a moderate drift.

**Figure 12:**
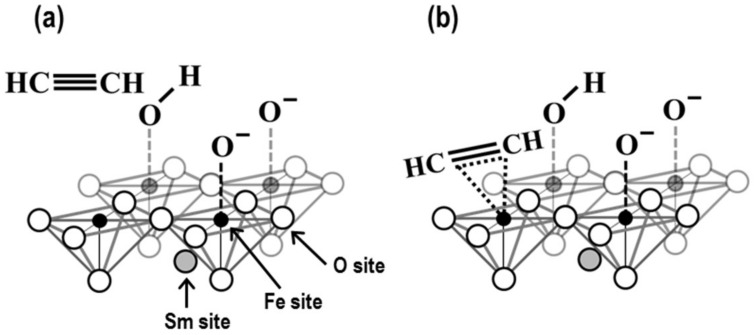
Schematic of the surface of perovskite-type oxide and appearance of adsorbed C_2_H_2_ on an active site of perovskite; (**a**) before and (**b**) after adsorption.

The electrical state of a B-site metal ion on a perovskite-type oxide might affect sensor properties because the sensor used for Fe-based perovskite-type oxides has shown a good response differentially [16]. The sensor response in this study might tentatively have progressed via chemisorbing C_2_H_2_ on an Fe^3+^ site, as shown in Figure 12. The Fe^3+^ of the SmFeO_3_ forms a six-coordinate complex in an octahedral-structured FeO_6_ and [(*t*_2g_)^3^(*e*_g_)^2^] in a high-spin state. Here, d*_x_^2^_-y_^2^* and d*_z_^2^* orbitals, which form a bond with oxygen, move into a high-energy state, and d*_xy_*, d*_yz_* and d*_xz_* orbitals, which have no bond, move into a low-energy state. Quintuple orbitals of Fe 3d eventually separate by forming octahedral FeO_6_. The Fe 4s orbital also loses an electron to lead to a vacant orbital by oxidizing from Fe^(0)^ to Fe^3+^. Therefore, the 3d and the 4s orbitals are HOMO and LUMO, respectively. On the other hand, the HOMO and LUMO of the C_2_H_2_ molecule are π and π* orbitals, respectively. To chemisorb C_2_H_2_ on Fe^3+^, it should be understood that an electronic interaction must occur between the HOMO and LUMO of each species [27]. When an electron that is evolved from an adsorbed oxygen is diminished by a hole in the SmFeO_3_ surface, this decreases conductivity of the oxide surface [28,29]. Therefore, it should be important for the SmFeO_3_ sensor device to have as much Fe^3+^ as possible on the surface [30].

**Figure 13:**
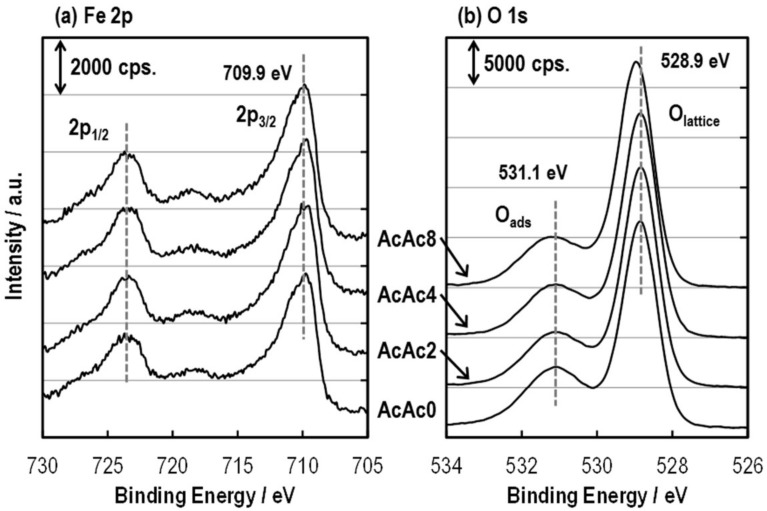
XPS spectra of the SmFeO_3_ thin-film device prepared from AcAc0, 2, 4 and 8 precursors sintered at 750 °C; (**a**) Fe 2p and (**b**) O 1s.

Figure 13 shows Fe 2p and O 1s spectra of the SmFeO_3_ thin-films of AcAc0, 2, 4 and 8. The Fe 2p_3/2_ showed a peak at 709.9 eV which corresponds to Fe^2+^ and Fe^3+^ cations. The O_1s_ also implies lattice oxygen and adsorbed oxygen at 528.9 and 531.1 eV, respectively. It might be found that there is no surface oxidation state change for each SmFeO_3_ thin-film. Table 1 summarizes the contents of various elements on the surfaces of the AcAcX thin-films. Both elements showed an increase and decrease from semi-quantitative analysis, and relativity was not obtained between the XPS results and the sensor responses to C_2_H_2_. Therefore, this could explain that the gas diffusion properties of the SmFeO_3_ thin-film improved the response to C_2_H_2_. Quantitative analyses and theoretical approaches are in progress to identify the sensing mechanism.

## 4. Conclusions

The impedancemetric C_2_H_2_ sensor was fabricated by using SmFeO_3_ thin-films prepared by a polymer precursor method. Crystalline and particle growth of the SmFeO_3_ thin-films could be controlled by the additive amount of AcAc in the solution with EG and metal nitrates. It could be illustrated by TG–DTA, FT–IR and UV–Vis that a high-coordination structure formed between metal cations and carboxylic oxygen. This affected the pyrolytic and sintering behaviors. The obtained SmFeO_3_ thin-films showed different responses to C_2_H_2_. Moreover, the SmFeO_3_ thin-film prepared by the AcAc8 precursor, which has good interface characteristics for a sensor, showed the best response to C_2_H_2_ by means of AC impedance.

## Figures and Tables

**Table 1:** Ratios of the elements on the surface of the SmFeO_3_ thin-film prepared from AcAc0, 2, 4 and 8 precursors sintered at 750 °C.

X in AcAcX	$\frac{Fe}{Sm+Fe}$	$\frac{O_{lat}}{Sm+Fe}$	$\frac{O_{ads}}{Sm+Fe}$	$\frac{O_{lat}+O_{ads}}{Sm+Fe}$
0	0.188	0.143	0.076	0.219
2	0.164	0.137	0.065	0.202
4	0.193	0.135	0.054	0.189
8	0.170	0.134	0.057	0.191
